# Exposure to low levels of photocatalytic TiO_2_ nanoparticles enhances seed germination and seedling growth of amaranth and cruciferous vegetables

**DOI:** 10.1038/s41598-022-23179-9

**Published:** 2022-10-29

**Authors:** Chi-Cheng Li, Sian-Ming Jhou, Yi-Chen Li, Jhih-Wei Ciou, You-Yen Lin, Shih-Che Hung, Jen-Hsiang Chang, Jen-Che Chang, Der-Shan Sun, Ming-Lun Chou, Hsin-Hou Chang

**Affiliations:** 1 Department of Hematology and Oncology, Buddhist Tzu Chi General Hospital, Hualien, Taiwan; 2 Center of Stem Cell & Precision Medicine, Hualien Tzu Chi Hospital, Hualien, Taiwan; 3 Tzu-Chi Senior High School Affiliated With Tzu-Chi University, Hualien, Taiwan; 4 Department of Molecular Biology and Human Genetics, Tzu-Chi University, Hualien, Taiwan; 5 Institute of Medical Sciences, Tzu-Chi University, Hualien, Taiwan; 6 Department and Graduate School of Computer Science, National Pingtung University, Pingtung, Taiwan; 7 Stella Maris High School, Hualien, Taiwan; 8 Department of Life Sciences, Tzu-Chi University, Hualien, Taiwan

**Keywords:** Nanoscale materials, Plant sciences

## Abstract

Titanium dioxide (TiO_2_) is one of the most common compounds on Earth, and it is used in natural forms or engineered bulks or nanoparticles (NPs) with increasing rates. However, the effect of TiO_2_ NPs on plants remains controversial. Previous studies demonstrated that TiO_2_ NPs are toxic to plants, because the photocatalytic property of TiO_2_ produces biohazardous reactive oxygen species. In contrast, another line of evidence suggested that TiO_2_ NPs are beneficial to plant growth. To verify this argument, in this study, we used seed germination of amaranth and cruciferous vegetables as a model system. Intriguingly, our data suggested that the controversy was due to the dosage effect. The photocatalytic activity of TiO_2_ NPs positively affected seed germination and growth through gibberellins in a plant-tolerable range (0.1 and 0.2 mg/cm^2^), whereas overdosing (1 mg/cm^2^) induced tissue damage. Given that plants are the foundations of the ecosystem; these findings are useful for agricultural application, sustainable development and maintenance of healthy environments.

## Introduction

Nanotechnology is an emerging technological advancement for the manipulation and synthesis of materials with a size range of 1–100 nm. Nanotechnology is a billion-dollar industry involving business and industrial investments that offer an array of tremendous applications^1,2^. Nanoparticles (NPs) (all abbreviations are available in the supplementary Table S1) can be categorized on the basis of their origin, such as natural, man-made, and engineered^3^. Environmental NPs have existed in nature from the beginning of Earth’s history and they are still found in the environment in the form of volcanic dust, lunar dust and mineral composites^2^. Meanwhile, thousands of tons of engineered NPs (ENPs) are produced annually, and they will inevitably be released into soil and waters and increase the load of ENPs in different environmental matrices, thereby attracting extensive attention on the potential impact of ENPs in the environment on aquatic and terrestrial organisms^3–8^. Once released in the environment, ENPs may contact or even enter plants, microorganisms and other organisms through the food chain. In particular, NPs have the potential to form highly reactive materials such as photocatalysts due to their highly reactive nature and large surface areas^2^.

Notably, among all NP categories, TiO_2_ is major type of NP produced globally^2^. TiO_2_ NPs are utilized in paint pigments, inks, papers, plastics and cosmetic sunscreens to provide protection against ultraviolet (UV) light^2,9^. In addition, natural and engineered TiO_2_ NPs have shown their potentials in various antimicrobial and biomedical applications^10–20^. As a result, scientific communities have paid extensive attentions to the interactions of TiO_2_ NPs with biological entities such as plants^10,11^. Highly sensitive to environmental factors, plants are vital life forms of all ecosystems and play a significant role in trophic transfer and the maintenance of worldwide ecological balance. Therefore, the exposure of plants to natural NPs or ENPs above particular levels may be toxic. The contact, interaction, accumulation and toxicity of NPs in plant systems are a recently formed field of research. After decades of studies, however, our current knowledge is not sufficient to formulate a detailed model of NP behavior and their fate in the environment^5^. For example, researchers have reported contradictory results that involve positive, negative and inconsequential effects from plants being exposed to TiO_2_ NPs^2^. From a toxicity perspective, previous studies have suggested that photocatalytic materials, such as TiO_2_ NPs, have negative impacts on terrestrial microorganisms and plants^2^. For instance, the associations of TiO_2_ NPs with the decrease of shoot biomass in wheat, delayed germination and root elongation in narbon bean (*Vicia narbonensis L*.) and *Zea mays L.*, and DNA damage in *Nicotiana tabacum* and *Allium cepa* have been reported^21–23^. By contrast, treatments of TiO_2_ NPs are associated with increases of light absorption and photosynthetic carbon reaction in spinach and maize and improvement of nitrogen photoreduction in soybeans and spinach^24–27^. These positive responses of plants led to the suggestion of using TiO_2_ NP as a nanofertilizer^26^. However, the concept of nanofertilizer is mainly focused on the nutrient supplemental role of NPs^26^, and whether the beneficial effect to plants contributes to the photocatalytic property of TiO_2_ NP remains elusive.

Despite these contradictory results, Ti and TiO_2_ are common and naturally occur in soil and volcanic ash^28–31^. Soil covering European surfaces contains an average 0.02–5.5% of TiO_2_^29^ and rocks in the USA contain approximately 1% of TiO_2_^30^. Theoretically, plants on Earth should have evolved to adapt to the existence of TiO_2_ in soil. Therefore, whether and how TiO_2_ NP may influence plant growth should be determined. In this study, we used the seed germination and growth of vegetables, *Amaranthus mangostanus*, *Brassica napus* and *Brassica rapa chinensis*, as model systems. We found that treatments of TiO_2_ NPs with low doses (0.1 and 0.2 mg/cm^2^) -induced enhancing effect, while treatments with a high dose (1 mg/cm^2^) displayed suppressive effect on seed germination and growth. The potential mechanism, applications and relationships with the photocatalytic properties are discussed.

## Materials and methods

### Chemicals and NPs

Chemicals such as N-acetylcysteine (NAC) were purchased from Sigma-Aldrich (St. Louis, MO, USA). UV light-responsive pure TiO_2_ NPs (Degussa P25; Evonik, Germany) were used as previously described^12,19,32^. The crystal structure of P25 TiO_2_ was a mixture of 75% anatase and 25% rutile TiO_2_. The purity was at least 99.5% TiO_2_ and primary particle size was 21 nm ± 10 nm, with a specific surface area of 50 ± 15 m^2^/g. Carbon-containing TiO_2_ NPs [TiO_2_(C)] were prepared using a sol–gel method^19,33^. The powders were subjected to calcination at 200 °C, and named C200; the detailed preparation of C200 has been reported elsewhere^19,33^. By photoreduction process using H_2_PtCl_6_ and TiO_2_ NPs as a platinum precursor and a pristine photocatalyst, respectively, platinum-containing TiO_2_ NPs [TiO_2_(Pt)] were prepared and characterized following previously described methods^13,15^. The NPs of zinc oxide (ZnO; < 40 nm)^9^ single-walled carbon nanotubes (CNTs), and silicon dioxide (SiO_2_; 10–20 nm) were purchased from Sigma–Aldrich. Nanodiamond (ND) NPs, with average sizes of 5 and 100 nm^9,34,35^, were purchased from Kay Diamond Products (Boca Raton, FL, USA).

### Seeds, soil and flowerpots

Seeds of vegetable *A. mangostanus*, *B. napus* and *B. rapa chinensis* were purchased from Sinon (Taichung, Taiwan). All seeds used this study were stored in an electronic dehumidifying dry cabinet (Taiwan Drytech, Taipei, Taiwan) with humidity < 40% before use, and then utilize as fresh as possible. Soil mixtures for cultivation and plastic flowerpots (diameter of 10 cm) were obtained from Green Orchids (Taipei, Taiwan). All experimental procedures are complied with national guidelines.

### Analysis of seed germination and seedling growth

To analyze the seed germination and seedling growth of *A. mangostanus*, *B. napus* and *B. rapa chinensis*, the soil surfaces of the pots were added without or with different amounts of NPs (0.1, 0.2 or 1 mg/cm^2^). Each flowerpot was seeded with 10–90 seeds depending on the experimental requirement. To avoid washing away the soil-surface TiO_2_, the plants were placed in a tray and absorb water (without TiO_2_) via capillary action through the holes in the bottom of the pot. After daily sunlight illumination and water supplements for 1 week, the germination rate and root and shoot length of the seedlings were examined and quantified. In cotton substrate experiments, sterile absorbent cotton (Taiwan Cotton, Taipei, Taiwan) was placed in sterile plastic cell culture dishes (diameter of 10 cm)^36,37^ with daily water supply to maintain the moisture. In experiments using antioxidant NAC, 100 µL of 1 mM NAC was added to each pot per day for 7 days. In experiments with visible light illumination, pots were illuminated by visible light (1 × 10^4^ lx) using light-emitting diode (LED) lamps (Philips Taiwan, Taipei, Taiwan) for 10 h/day for 7 days; at the same time, UV cut-off filters (400 nm; Edmund Optics, Barrington, NJ, USA)^32^ were used to prevent the illumination of small fractions with UV wavelength. A light meter (model LX-102; Lutron Electronic Enterprises, Taiwan)^14,16^ was used to examine the illumination density.

### Detection of plant hormone gibberellins (gibberellic acids; GAs)

One week old seedlings of *A. mangostanus* treated with or without TiO_2_ NPs and the antioxidant NAC were harvested. The samples were freshly prepared before enzyme-linked immunosorbent assay (ELISA) examinations. GA extraction and purification prior to immunoassay were conducted according to previous reports^38,39^. The homogenized samples were extracted in 80% cold (v/v) aqueous methanol solution overnight at 4 °C (with 10 mg/L butylated hydroxytoluene to prevent oxidation). The supernatants were collected after centrifugation at 10,000×*g* (4 °C) for 20 min to remove the insoluble debris. The crude extract was passed through a 0.45 µm filter (Merck Millipore, Billerica, MA, USA). A 400-μl aliquot of the filtrate was dried under vacuum using a SpeedVac vacuum concentrators (Thermo Fisher Scientific, Waltham, MA, USA). The extraction residues were dissolved, diluted in phosphate-buffered saline (PBS) (0.01 M, pH 9.2), and adjusted to pH 8.5. The levels of GAs were detected using an ELISA kit purchased from MyBioSource (San Diego, CA, USA).

### Electron microscopy

Transmission electron microscopy (TEM) and scanning electron microscopy (SEM) examinations of TiO_2_ treated seedlings and TiO_2_ NPs were performed using a Hitachi H-7500 TEM (Hitachi, Tokyo, Japan) and Hitachi S-4700 SEM (Hitachi)^13,15,19,32,40^, respectively. The seedlings were pre-fixed with 2.5% glutaraldehyde in 0.1 M phosphate buffer (pH 7.3) for 1 h. The seedling samples were washed with 0.1 M PBS twice at a 10-min interval. The samples were post-fixed with 1% osmium for 1 h and washed with 5% sucrose. The seedling samples were dehydrated in a graded ethanol-acetone series, embedded in Spurr’s resin (Electron Microscopy Sciences, Hatfield, PA, USA), and sliced using a Ultracut-R ultramicrotome (Leica, Wetzlar, Germany). The seedling tissue sections were finally immobilized on single-well copper grids for TEM analysis.

### Statistical analysis

All results were calculated from data obtained from three independent experiments. Analysis of variance (ANOVA) was used to assess the statistical significance of differences results. The significance of the data was examined using one-way ANOVA, followed by the post hoc Bonferroni-corrected t test. A probability of type 1 error (α = 0.05) was recognized as the threshold for statistical significance. The statistical tests were carried out, and graphed using Microsoft Excel (Microsoft Taiwan, Taipei, Taiwan) and SigmaPlot (Systat Software, Point Richmond, CA, USA) software as previously described^41^.

## Results

### TiO_2_ NPs markedly enhanced the seed germination and seedling growth of ***A. mangostanus***

**Figure 1:**
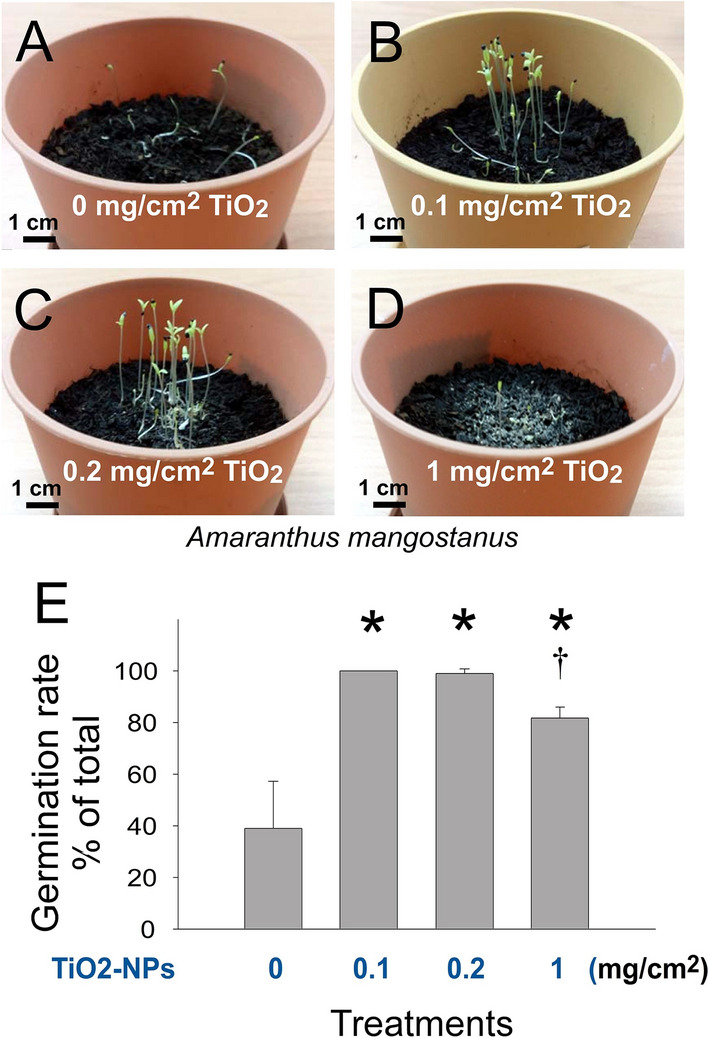
TiO_2_ NPs treatments on soil enhanced the germination rate of *Amaranthus mangostanus* seeds. Images of 1-week-old seedlings of *Amaranthus mangostanus* after seeding on soil without (**A**) or with (**B–D**) addition of pure TiO_2_ NPs are shown. The seed germination rates of aforementioned conditions were quantified (**E**). *P < 0.05 vs. 0 mg/cm^2^ TiO_2_ untreated groups, †*P* < 0.05 vs. 0.2 mg/cm^2^ TiO_2_ groups. n = 3 (3 independent experiments; each experiment with 30 seeds). (**A–D**) Pot diameter: 10 cm; scale bars: 1 cm.

*Amaranthus* is a cosmopolitan genus with species cultivated as leaf vegetables *A. mangostanus* was used in this study because of its small seeds, fast growth rate and potential application in agriculture. Under UV irradiation, the photocatalytic activity of anatase TiO_2_ nanoparticles (NPs) releases reactive oxygen species (ROS) and enables TiO_2_ NPs to serve as biocides^12,42–44^. Prior to the experiments, we hypothesized that the addition of TiO_2_ NPs may exert a negative impact on the seed germination and growth of *A. mangostanus*. Intriguingly, we found that supplemention of TiO_2_ NPs on the soil markedly enhanced the seed germination rate of *A. mangostanus* in all three dosages within 1 week (Fig. 1A–D, representative images; Fig. 1E, TiO_2_ untreated vs. TiO_2_ treated groups, *P < 0.05).

**Figure 2:**
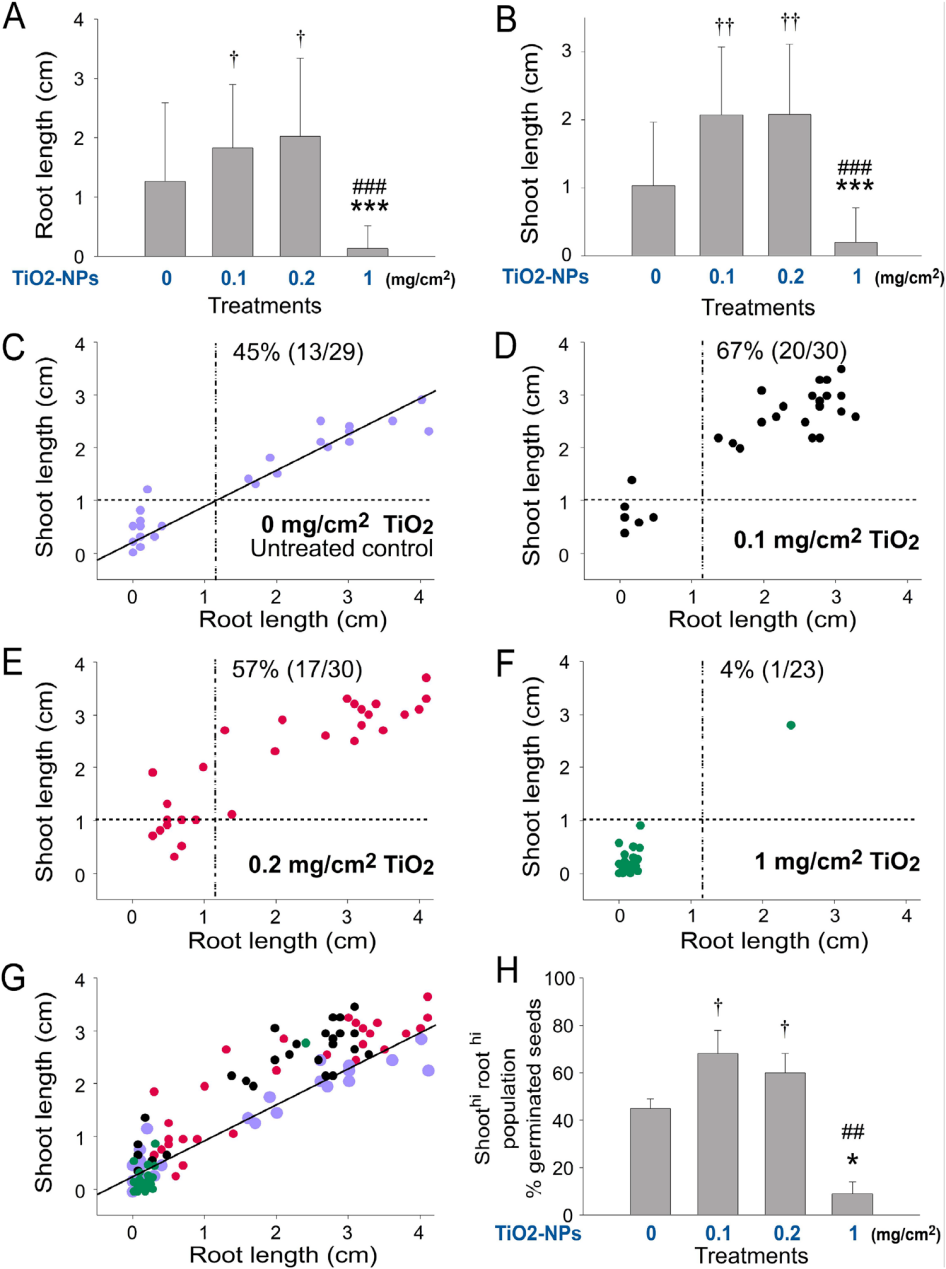
Soil TiO_2_ NP levels affected the seedling growth of *Amaranthus mangostanus.* Analyses of root (**A**) and shoot (**B**) length of 1-week-old *Amaranthus mangostanus* seedlings. Root–shoot 2D graphs of the seedling without (**C**) and with different doses of TiO_2_ NP treatments (**D–F**), and an overlay (**G**) are shown. The regression lines of untreated groups were indicated (**C**,**G**). The shoot^hi^ root^hi^ population (upper-right quadrant) of seedlings in each condition was quantified (**H**). The vertical and horizontal dotted lines in (**C–F**) are the mean values of root length and shoot length of the untreated control groups (**C**), respectively. †*P* < 0.05, ††*P* < 0.01, *P < 0.05, ***P < 0.001 vs. 0 mg/cm^2^ TiO_2_ untreated groups; ##*P* < 0.01, ###*P* < 0.001 vs. respective 0.2 mg/cm^2^ TiO_2_ groups. n = 90 (3 independent experiments; each experiment with 30 seeds) (**A,B**). n = 30 (3 independent experiments; each experiment with 10 seeds) (**C–G**). n = 3 (3 independent experiments; each experiment with 30 seeds) (**H**).

The high shoot length in the TiO_2_ groups (Fig. 1A vs. Fig. 1B,C), suggested that TiO_2_ NPs may also increase the growth rate of the seedlings. Further quantitative analyses of the length of shoot and root of seedlings revealed that TiO_2_ NPs markedly enhanced the shoot and root growth of the seedlings in low-dose treatments (Fig. 2A,B, 0 vs. 0.1 and 0.2 mg/cm^2^ groups; root analyses, †*P* < 0.05; shoot analyses, ††*P* < 0.01), but suppressed seedling growth at high dose treatments (Fig. 2A,B, 0 vs. 1 mg/cm^2^; groups, root and shoot analyses, ***P < 0.001). When the shoot length and root length of the control group (0 mg/cm^2^ TiO2 NPs) were plotted in a 2-dimensional (2D) graph with regression (solid) and averaged shoot and root length (dotted) lines, we found that approximately 45% of the seedlings were located at the shoot^hi^ and root^hi^ areas (Fig. 2C, upper right quadrant, a well-grown seedling population), and the seedlings were equally distributed at both sides of the regression line (Fig. 2C, untreated control, 0 mg/cm^2^ group). When we applied the aforementioned dotted lines (averaged shoot and root length of normal) to the TiO2 NP-treated conditions, the 2D graphs revealed that two low-dose groups showed a higher percentage of shoot^hi^ root^hi^ population (67 and 57% in Fig. 2D,E, respectively), whereas the high dose group showed a lower percentage of shoot^hi^ root^hi^ population (4%, Fig. 2F). These results were in agreement with the shoot and root length analyses (Fig. 2A,B). Notably, when we overlaid these 2D graphs (Fig. 2C–F), we found that the shoot^hi^ root^hi^ population in the TiO_2_ groups was almost exclusively located at the upper area above the regression line of the normal control (Fig. 2G), suggesting that the growth enhancement effect was primarily mediated through the induction of shoot growth. Consistently, quantitative analyses further indicated that the shoot^hi^ root^hi^ populations were markedly increased in the two low-dose (Fig. 2H, 0 vs. 0.1 and 0.2 mg/cm^2^ groups; †*P* < 0.05) TiO_2_ groups, whereas the shoot^hi^ root^hi^ populations were markedly suppressed in the high-dose TiO_2_ groups, when compared with untreated controls (Fig. 2H, 0 vs. 1 mg/cm^2^ groups; **P < 0.01).

### ZnO NPs markedly enhanced the seed germination and seedling growth of *A. mangostanus*

**Figure 3:**
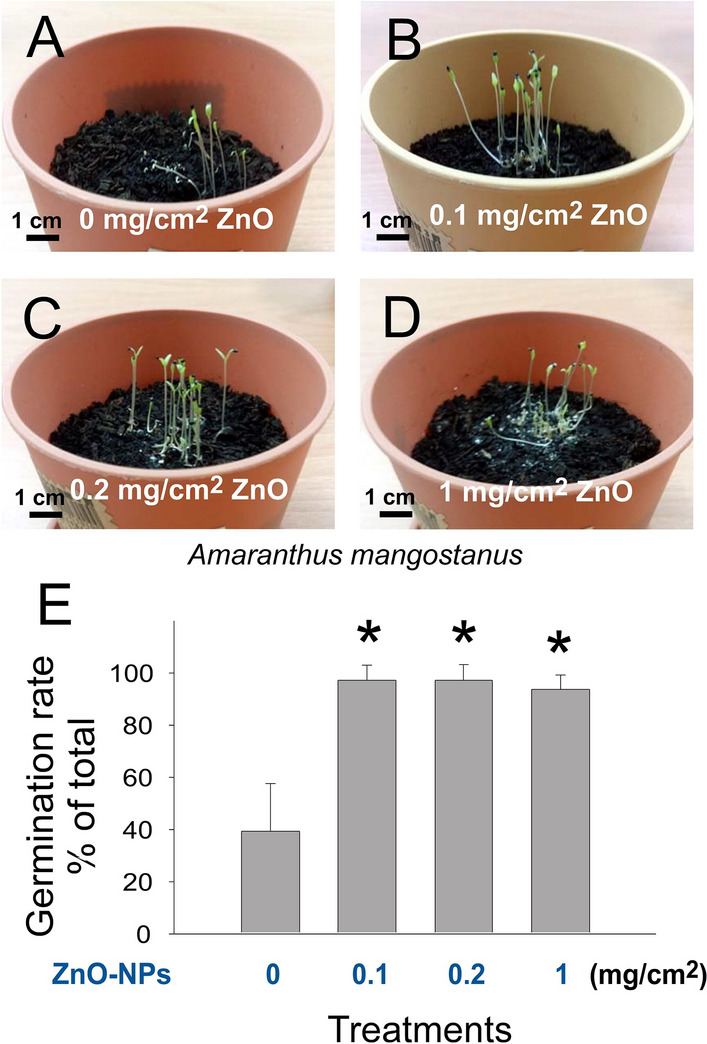
Treatments of ZnO NPs on soil enhanced the germination rate of *Amaranthus mangostanus* seeds. Images of 1-week-old seedlings of *Amaranthus mangostanus* after seeding on soil without (**A**) or with (**B–D**) the addition of ZnO NPs are shown. The seed germination rates of aforementioned conditions were quantified (**E**). *P < 0.05 vs. 0 mg/cm^2^ ZnO untreated groups. n = 3 (3 independent experiments; each experiment with 30 seeds). (**A–D**) Pot diameter: 10 cm; scale bars: 1 cm.

**Figure 4:**
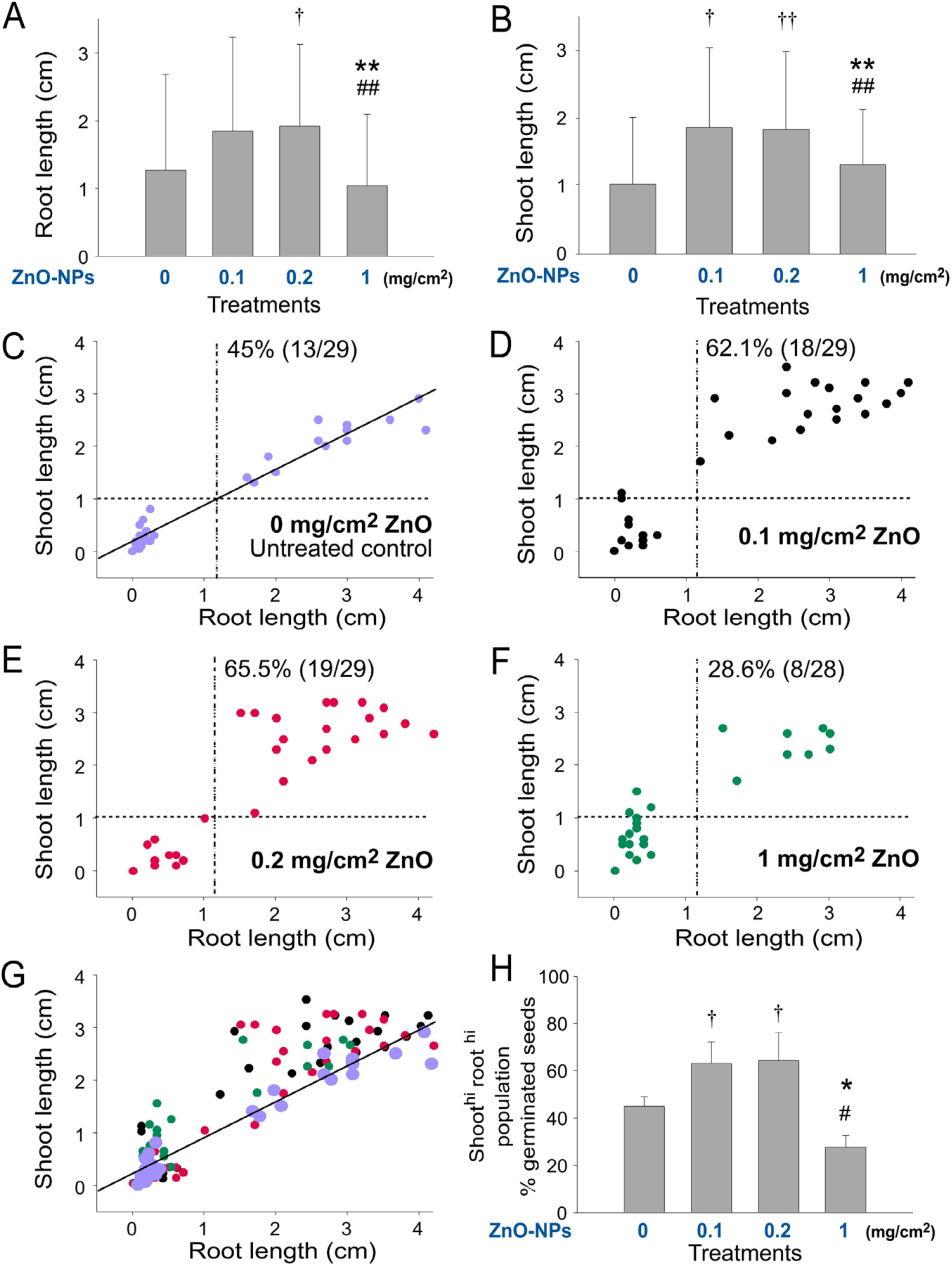
Soil ZnO NP levels affect the seedling growth of *Amaranthus mangostanus.* Analyses of root (**A**) and shoot (**B**) length of 1-week-old *Amaranthus mangostanus* seedlings. Root–shoot 2D graphs of the seedling without (**C**) and with different doses of ZnO NP treatments (**D–F**), and an overlay (**G**) are shown. The regression lines of untreated groups were indicated (**C,G**). The shoot^hi^ root^hi^ population (upper-right quadrant) of seedlings in each condition was quantified (**H**). The vertical and horizontal dotted lines in (**C–F**) are the mean values of root length and shoot length of the untreated control groups (**C**), respectively. †*P* < 0.05, ††*P* < 0.01, *P < 0.05, **P < 0.01 vs. 0 mg/cm^2^ ZnO untreated groups; #*P* < 0.05, ##*P* < 0.01 vs. respective 0.2 mg/cm^2^ ZnO groups. n = 90 (3 independent experiments; each experiment with 30 seeds) (**A,B**). n = 30 (3 independent experiments; each experiment with 10 seeds) (C–G). n = 3 (3 independent experiments; each experiment with 30 seeds) (**H**).

TiO_2_ NP is a UV-responsive photocatalyst. To investigate whether the addition of other photocatalytic NPs to the soil is still sufficient to achieve similar effects, ZnO NP, another photocatalytic material^45^, was employed. Following the same approaches in TiO_2_ experiments, we found that supplemention of ZnO NPs to the soil also markedly increased the seed germination rate and growth of *A. mangostanus* (Fig. 3A–D, representative images; Fig. 1E, TiO_2_ untreated vs TiO_2_ treated groups, *P < 0.05). In addition, root and shoot length analyses revealed that ZnO NPs markedly increased the growth of both root and shoot of the seedlings (Fig. 4A,B, 0 vs. 0.2 mg/cm^2^ groups; root analyses, †*P* < 0.05; shoot analyses, ††*P* < 0.01), but suppressed seedling growth at high dose treatments (Fig. 4A,B, 0 vs. 1 mg/cm^2^; groups, root and shoot analyses, **P < 0.01). Shoot–root 2D graphs also revealed that ZnO NP treatments markedly enhanced the shoot^hi^ root^hi^ populations of the two low-dose groups (Fig. 4H, 0 vs. 0.1 and 0.2 mg/cm^2^ groups; †*P* < 0.05), but suppressed the shoot^hi^ root^hi^ population in the high-dose group (Fig. 4H, 0 vs. 1 mg/cm^2^ groups; *P < 0.05). The shoot^hi^ root^hi^ population in the ZnO groups was exclusively located at the upper region above the regression line of normal control (Fig. 4G), suggesting that the growth enhancement effect was primarily mediated through the induction of shoot growth.

### TiO_2_ NPs markedly enhanced the seed germination and seedling growth of ***A. mangostanus*** on sterilized cotton in Petri dishes

**Figure 5:**
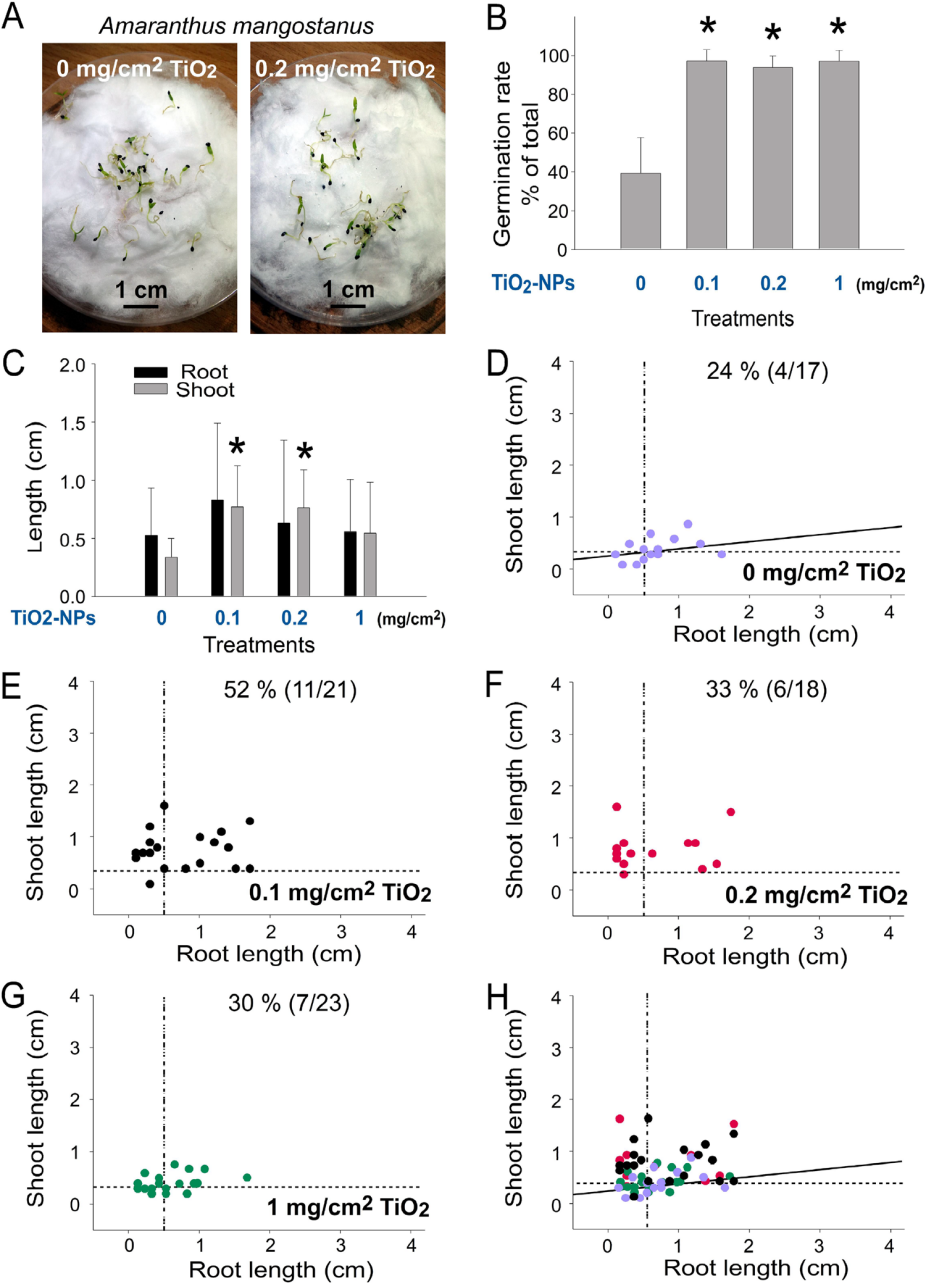
TiO_2_ NP levels of sterilized cotton substrates positively affect the germination rate of *Amaranthus mangostanus* seeds. Analyses of image (**A**), seed germination rate (**B**) and root and shoot length (**C**) of 1-week-old *Amaranthus mangostanus* seedlings. Root–shoot 2D graphs of the seedlings without (**D**) and with different doses of TiO_2_ NP treatments (**E–G**), and an overlay (**H**) are shown. The vertical and horizontal dotted lines in (**D–H**) are the mean values of root length and shoot length of the untreated control groups (**D**), respectively. *P < 0.05 vs. 0 mg/cm^2^ TiO_2_ untreated groups. n = 3 (3 independent experiments; each experiment with 30 seeds) (**B**). n = 90 (3 independent experiments; each experiment with 30 seeds) (**C**). n = 30 (3 independent experiments; each experiment with 10 seeds) (**D–H**). Scale bars: 1 cm (**A**).

As mentioned earlier, TiO_2_ NP exerts photocatalytic property and can be used as a bactericidal agent when exposed to solar UV light. Soil microbiota has been demonstrated to play a critical role in plant survival and growth^46,47^. The antibacterial property of photocatalytic NPs has been suggested to influence plant growth^48,49^. To investigate whether the aforementioned property of TiO_2_ NP on the enhancement of seed germination and growth is mediated through sterilization of soil bacteria, sterilized cotton and Petri dishes were employed as culture substrates. Following the same approach, we found that TiO_2_ NP exerted enhancing property on the seed germination and growth of *A. mangostanus* on sterilized substrates (Fig. 5A representative images, Fig. 5B, germination rate, 0 vs. 0.1, 0.2 and 1 mg/cm^2^ groups; *P < 0.05; Fig. 5C, seedling shoot growth, 0 vs. 0.1 and 0.2 mg/cm^2^ groups; *P < 0.05), with the typical preferential enhancement of shoot growth (Figs. 5D–H, 2D graphs; 5H, shoot^hi^ root^hi^ population mainly located at the upper region above the regression line). Because sterilized-cotton substrates preserved the positive effect of TiO_2_-NPs, these results suggested that the plant enhancing property was not mediated through the antibacterial property of TiO_2_ NP. The seedlings grown on cotton had a relatively smaller size than the seedlings grown in soil, possibly because cotton lacks critical nutrients found in soil (Fig. 2A,B vs. Fig. 5C).

### NPs without photocatalytic activity cannot exert the enhancing effect on *A. mangostanus *seeds

**Figure 6:**
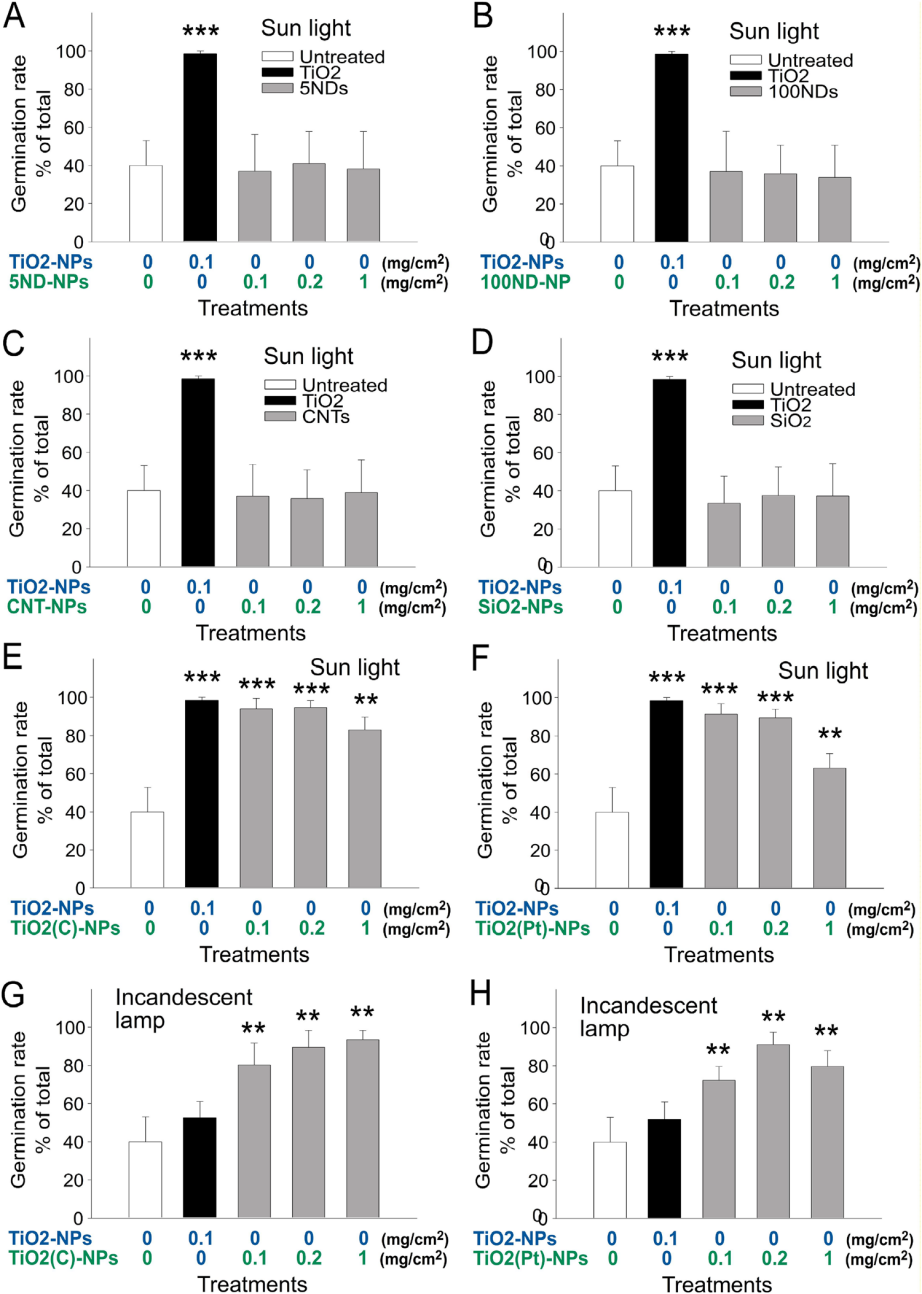
Photocatalysis is essential for TiO_2_ NP-mediated enhancement of seed germination. The germination rates of *Amaranthus mangostanus* seeds grown on soil with supplements of 5 nm nanodiamond (ND) (**A**), 100 nm ND (**B**), carbon nanoparticle (NP) (**C**), SiO_2_ NP (**D**), visible light-responsive photocatalysts TiO_2_ (**C,E,G**) and TiO_2_(Pt) (**F,H**) under daily sunlight (**A–F**) or visible light (**G,H**) illumination were compared with pure TiO_2_ (UV-responsive photocatalyst) and then quantified. **P < 0.01, ***P < 0.001 vs. 0 mg/cm^2^ TiO_2_ untreated groups. n = 3 (3 independent experiments; each experiment with 30 seeds).

A previous report suggested that NPs may have direct enhancing property (with or without inducing photocatalytic reaction) on the stimulation of plant growth^50–52^. Four NPs, namely 5 nm ND (5ND-NP), 100 nm ND (100ND-NP), single-walled CNT NP (CNT-NP) and silicon dioxide NP (SiO_2_-NP), were employed to investigate whether those non-photocatalytic NPs are sufficient to enhance seed germination and growth. Compared with TiO_2_ NP, these four types of NPs did not exert considerable enhancing effects on the seed germination (Fig. 6A–D). To further investigate the involvement of photocatalytic property, we employed two visible light responsive photocatalysts, carbon-containing TiO_2_ NP [TiO_2_(C)] and platinum-containing TiO_2_ NP [TiO_2_(Pt)]^13,15,18,19,33^, and was compared their performance with the pure TiO_2_ NP (UV-responsive photocatalyst; Fig. 6E–H). Under UV irradiation, pure TiO_2_, TiO_2_(C) and TiO_2_(Pt) NPs all exert photocatalytic properties (Fig. 6E,F; untreated vs. TiO_2_-treated groups, **P < 0.01, ***P < 0.001). By contrast, under visible light (incandescent lamp) illumination, only TiO_2_(C) and TiO_2_(Pt) NPs can exert photocatalytic property^13,19^, while pure TiO_2_ NP cannot^12,19^. In this study, we found that pure TiO_2_, TiO_2_(C), and TiO_2_(Pt) NPs all exerted seedling enhancing property under sunlight (Fig. 6E,F; containing UV), whereas only TiO_2_(C) and TiO_2_(Pt) NPs but not pure TiO_2_ exerted enhancing property under visible light illumination (Fig. 6G,H; untreated vs. TiO_2_-treated groups, **P < 0.01). These results suggested that the photocatalysis reaction is essential for the aforementioned NPs to stimulate seed germination.

### The enhancing property is associated with ROS-induced down-regulation of the growth hormone gibberellins

**Figure 7:**
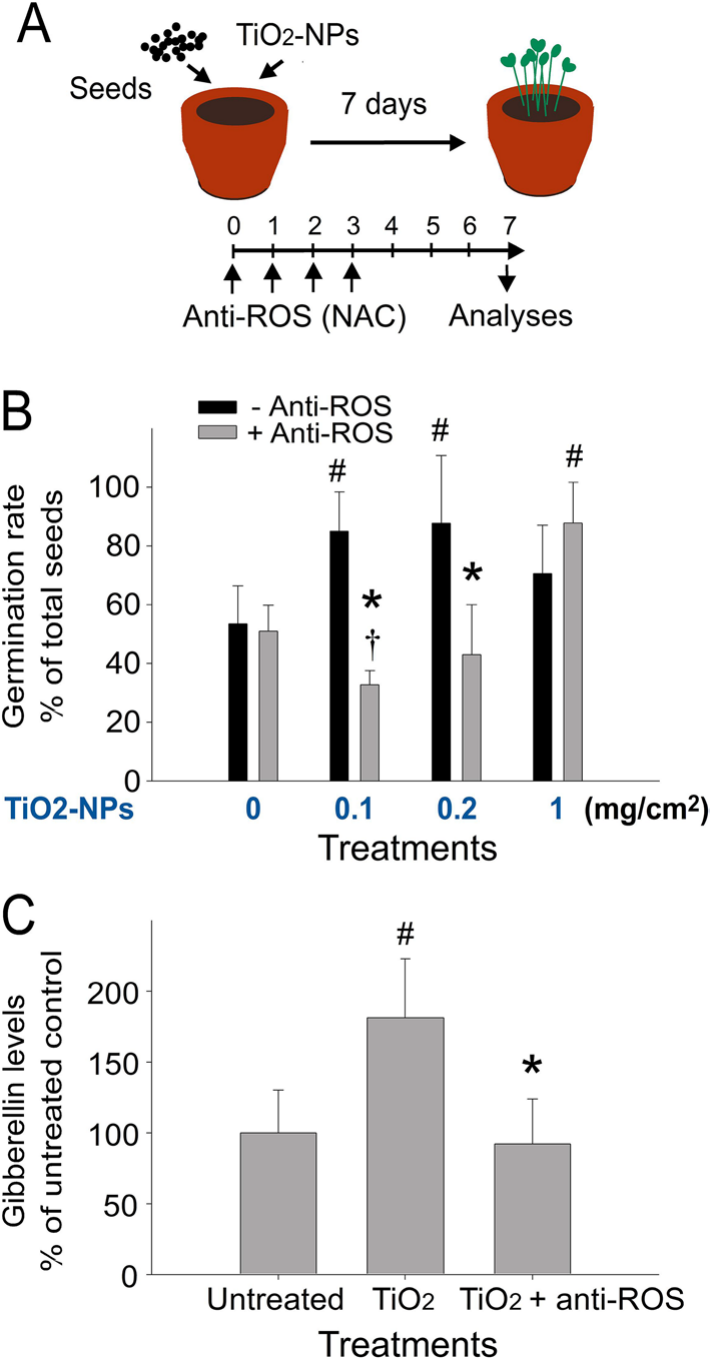
TiO_2_ NP-mediated enhancement on the seed germination is associated with ROS-modulated levels of the plant hormone gibberellins. Experimental outline (**A**), the seed germination rate with or without anti-ROS agent NAC treatments (**B**) and the expression levels of plant hormone gibberellins (**C**) are showed. #*P* < 0.05 vs. 0 mg/cm^2^ TiO_2_ untreated groups (**B,C**); *P < 0.05 vs. respective without anti-ROS treatment groups (-anti-ROS) (B); †*P* < 0.05 vs. TiO_2_ groups. n = 3 (3 independent experiments; each experiment with 30 seeds) (B); n = 3 (3 independent experiments; each experiment with 80 seeds).

To further investigate whether such an enhancing effect on seed germination is mediated through photocatalysis produced ROS, antioxidant NAC treatment was employed. We found that NAC treatment markedly reversed TiO_2_—induced enhancement of seed germination (Fig. 7A experiment outline; Fig. 7B, TiO_2_-untreated vs. TiO_2_-treated groups, #*P* < 0.05; NAC-untreated vs. NAC-treated groups, *P < 0.05). ROS can regulate levels of plant growth hormone gibberellins (GAs)^53^, and GAs stimulate shoot growth^54–56^. Given that ROS (Fig. 7B) and shoot growth (Figs. 2, 4) are two phenomena associated with TiO_2_ NP-enhanced seed germination and growth, we hypothesized that GA may be involved in growth regulation in this model. In agreement with our suggestion, the analysis results revealed that the suppression effect of NAC was associated with the suppression of TiO_2_-induced elicitation of GAs in the seedlings (Fig. 7C, TiO_2_-untreated vs. TiO_2_-treated groups, #*P* < 0.05; NAC-untreated vs. NAC-treated groups, *P < 0.05).

### The enhancing property of TiO_2_ NPs can also be applied to 2 other plants ***B. napus ***and ***B. rapa chinensis***

**Figure 8:**
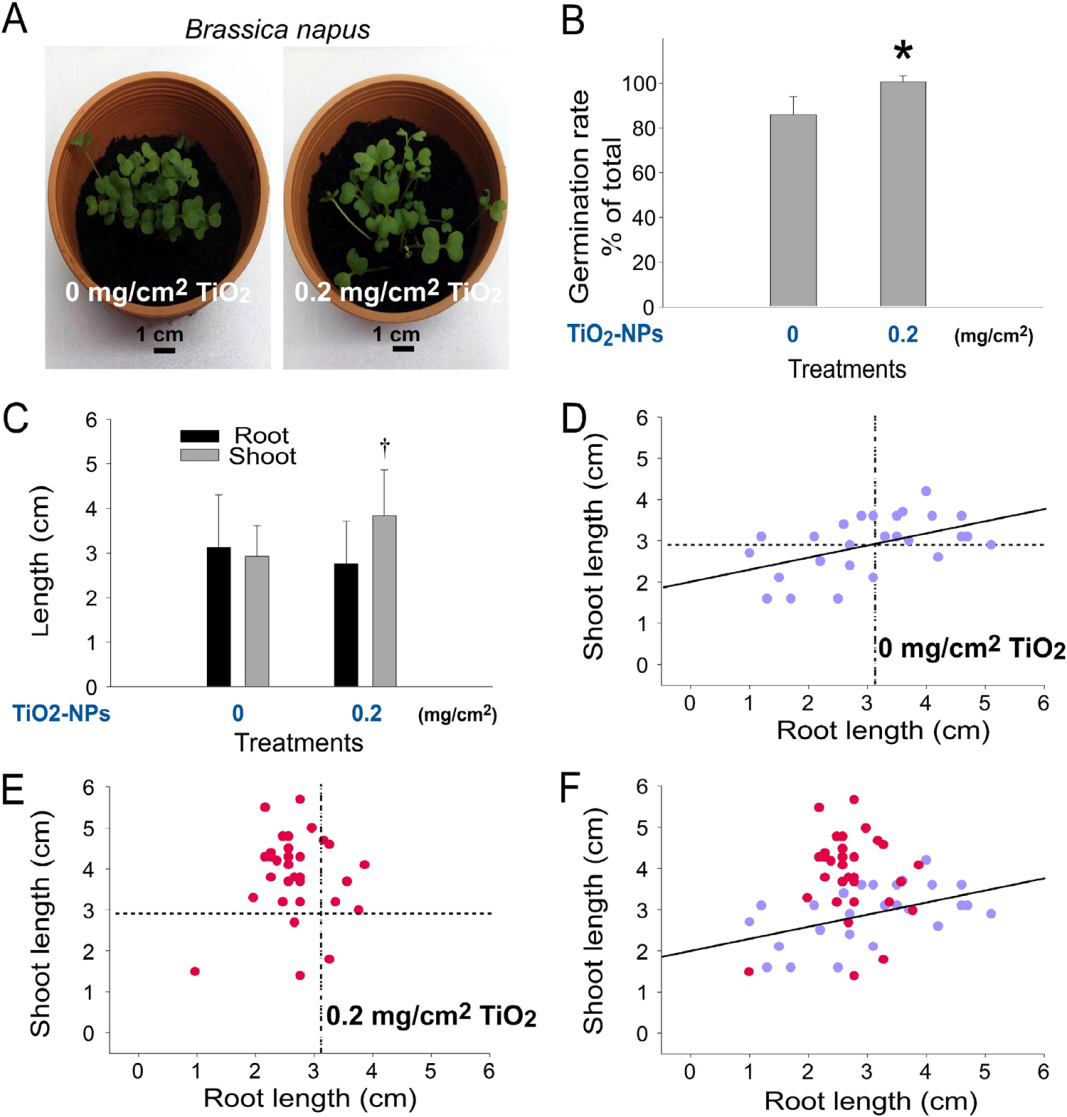
TiO_2_ NP-mediated enhancement on the seed germination and growth of soil can also be applied to *Brassica napus.* Analyses of image (**A**), seed germination rate (**B**) and root and shoot length (**C**) of 1-week-old *Brassica napus* seedlings. Root–shoot 2D graphs of the seedlings without (**D**) and with TiO_2_ NP treatments (**E**), and an overlay (**F**) are shown. The vertical and horizontal dotted lines in (**D,E**) are the mean values of root length and shoot length of the untreated control groups (**D**), respectively. *P < 0.05, †*P* < 0.05 vs. 0 mg/cm^2^ TiO_2_ untreated groups. n = 3 (3 independent experiments; each experiment with 30 seeds) (**B**). n = 90 (3 independent experiments; each experiment with 30 seeds) (**C**). n = 30 (3 independent experiments; each experiment with 10 seeds) (D-F). (**A**) Pot diameter: 10 cm; scale bar: 1 cm.

**Figure 9:**
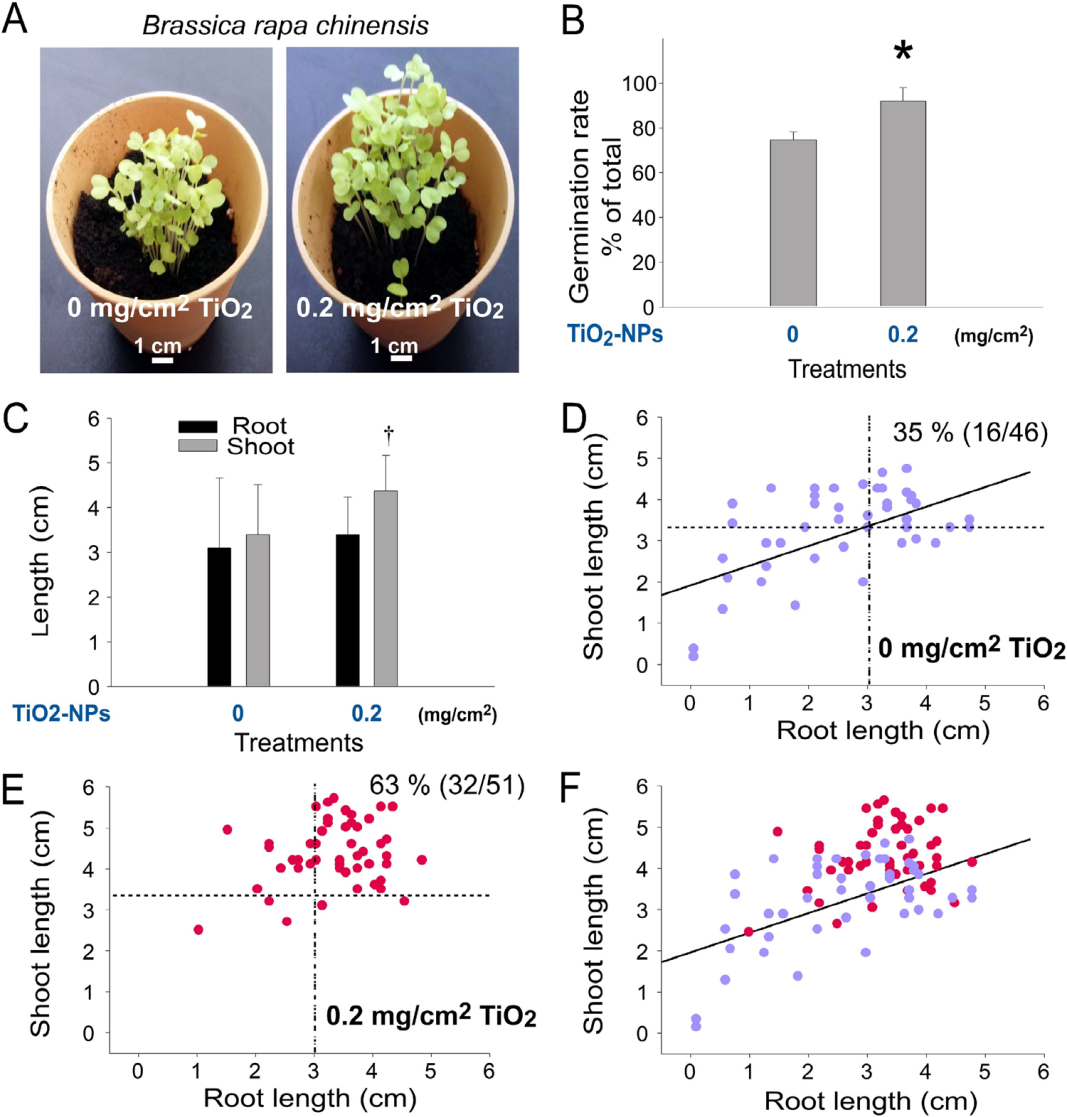
TiO_2_ NP-mediated enhancement on the seed germination and growth of soil can also be applied to *Brassica rapa chinensis*. Analyses of image (**A**), seed germination rate (**B**) and root and shoot length (**C**) of 1-week-old *Brassica rapa chinensis* seedlings. Root–shoot 2D graphs of the seedlings without (**D**) and with TiO_2_ NP treatments (**E**), and an overlay (**F**) are shown. The vertical and horizontal dotted lines in (**D,E**) are the mean values of root length and shoot length of the untreated control groups (**D**), respectively. *P < 0.05, †*P* < 0.05 vs. 0 mg/cm^2^ TiO_2_ untreated groups. n = 3 (3 independent experiments; each experiment with 30 seeds) (**B**). n = 90 (3 independent experiments; each experiment with 30 seeds) (**C**). n = 30 (3 independent experiments; each experiment with 10 seeds) (**D–F**). (**A**) Pot diameter: 10 cm; scale bar: 1 cm.

To investigate whether the seedling enhancing property of TiO_2_ NPs can also be applied to other plants, seeds of two cruciferous vegetables *B. napus* and *B. rapa chinensis* were employed. Here we found that such an enhancing effect of TiO_2_ NP could indeed be applied to *B. napus* (Fig. 8A, representative images; Fig. 8B, germination rate, TiO_2_-untreated vs. TiO_2_-treated groups *P < 0.05; Fig. 8C, seedling shoot growth, TiO_2_-untreated vs. TiO_2_-treated groups ††*P* < 0.01; Figs. 8D–F, 2D graphs) and *B. rapa chinensis* (Fig. 9A, representative images; Fig. 9B, germination rate, TiO_2_-untreated vs. TiO_2_-treated groups *P < 0.05; Fig. 9C, seedling shoot growth, TiO_2_-untreated vs. TiO_2_-treated groups +  + *P* < 0.01; Figs. 9D–F, 2D graphs), with its featured shoot-preferential enhancement (Figs. 8C,9C, TiO_2_-untreated vs. TiO_2_-treated groups, ††*P* < 0.01).

### High—dose TiO_2_ NP—induced tissue damage of seedlings

**Figure 10:**
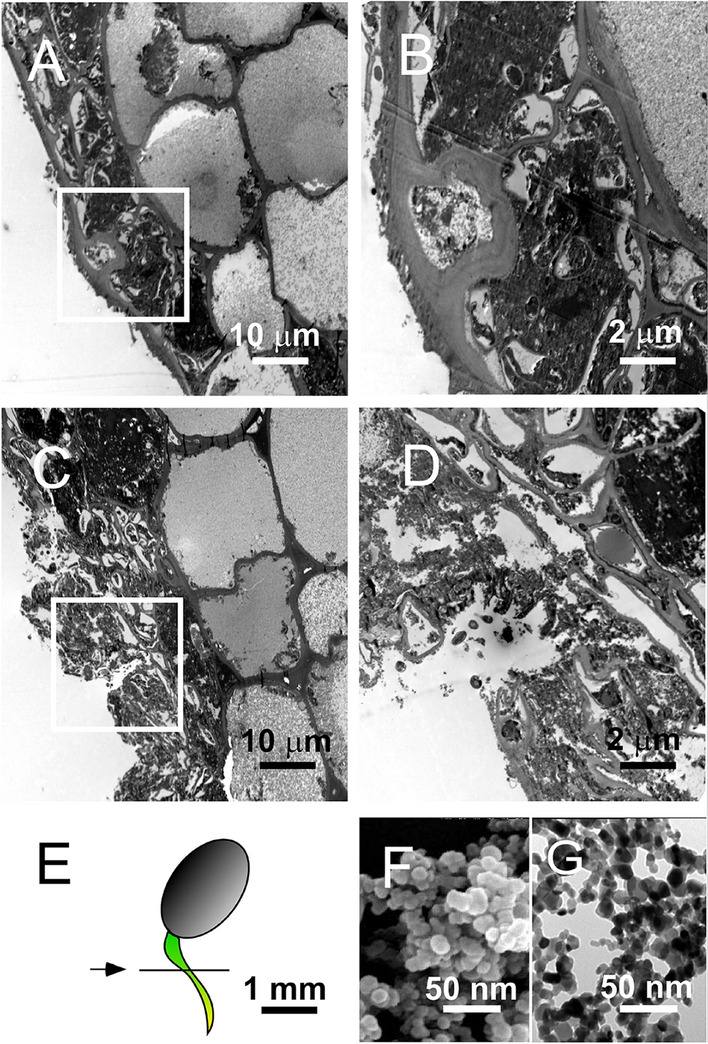
Electron microscopy analysis of high level TiO_2_ NP-induced tissue damages on seedlings of *Amaranthus mangostanus.* TEM images of root tissue samples of seedlings *Amaranthus mangostanus* in 0 (**A,B**) and 1 mg/cm^2^ TiO_2_ groups (**C,D**), at low (**A,C**) and high (**B,D**) magnifications are shown. The white square highlighted areas in (**A,C**) are shown at a higher magnification in (**B,D**), respectively. The section position in (**A–D**) is indicated in (**E**). The SEM and TEM images of TiO_2_ NPs are also shown in (**F**) and (**G**), respectively. Scale bars: (**A,C**) 10 µm; (**B,D**) 2 µm; (**E**) 1 mm; (**F,G**) 50 nm.

Although high dose treatments of TiO_2_ NPs exert enhancing properties on seed germination (Fig. 1), the seedling growth was also markedly suppressed (Fig. 2). Using TEM, we found that high dose treatments of TiO_2_ NPs induced considerable tissue damage on the outer layer of seedling samples (Fig. 10A,B, vs. Fig. 10C,D: representative images of normal vs. 1 mg/cm^2^ TiO2-treated; Fig. 10E, tissue section position of seedling; Fig. 10F,G, SEM and TEM images of TiO_2_ NPs), suggesting that photocatalysis produced ROS by high—dose TiO_2_ NPs was harmful to the seedlings.

## Discussion

TiO_2_ NPs-mediated negative impacts on plants have been reported in various studies^21–23^. By contrast, TiO_2_ NPs have also been shown to enhance plant growth^26^, whether TiO_2_ NP–mediated photocatalysis is involved remains uncertain. The controversy has confused researchers for years^2^. In this present study, our data suggested that such controversial properties of TiO_2_ NPs on plant growth were likely due to the dosage effect of TiO_2_ NPs; low dose (0.1, 0.2 mg/cm^2^) was beneficial, while high dose (1 mg/cm^2^) was harmful (Figs. 1–2). Such positive effect primarily involves TiO_2_ NP-mediated enhancement of germination rate and seeding growth (Figs. 1–2). Intriguingly, similar effect also observed in the ZnO treatments (Figs. 3–4). Because illumination was essential to enhance seed germination and seedling growth (Fig. 6), these results collectively suggest that the photocatalysis reaction is critical in for the photocatalytic NP-mediated enhancement. Evidences revealed that TiO_2_ displayed higher photocatalytic activities compared to ZnO under UV illumination^57,58^. Consequently, it is reasonable to observe an obvious suppressive effect on the seed germination in the high dose (1 mg/cm^2^) TiO_2_-treatments (Fig. 1E, †*P* < 0.05; 0.2 mg/cm^2^ vs. 1 mg/cm^2^ groups), as compared to the high dose (1 mg/cm^2^) ZnO-treatments (Fig. 3E, no obvious suppression 0.2 mg/cm^2^ vs. 1 mg/cm^2^ groups).

Previous studies have suggested that the small-size of natural NPs enable the positive impact on the plants^59^. However, here we found that treatments of TiO_2_ NPs are not sufficient to conduct such enhancement unless illumination with proper wavelength was provided [Fig. 6A–F vs. Fig. 6G,H, TiO_2_ groups; 6G and 6H, TiO_2_ vs. TiO_2_(C) and TiO_2_(Pt) groups]. This suggested that the treatment of NPs alone is insufficient to stimulate seed germination and growth, and the induction of photocatalysis is essential. The treatments of antioxidant NAC could reverse TiO_2_ NP-mediated enhancement (Fig. 7B), further indicating the involvement of photocatalysis- produced ROS is involved. The enhancing effect of TiO_2_ NPs and the suppressive effect of NAC on seed germination are associated with increased and suppressed GA levels, respectively (Fig. 7C), which further suggests the involvement of ROS in GA regulation.

The TiO_2_ NP-mediated photocatalytic reaction produces strong reducing and oxidizing electrons and electron-vacancy holes^12^. These electrons and holes can react with atmospheric water and oxygen (H_2_O and O_2_) to yield reactive oxygen species (ROS), such as hydroxyl radicals (^●^ OH), superoxide anions (O_2_^−^), and hydrogen peroxide (H_2_O_2_), products are extremely reactive when in contact with organic compounds^12^. Treatments of TiO_2_ NP is a feasible model to observe the impacts of exogenous ROS, as the effect of exogenous ROS on seed germination have not yet been extensively studied. A detailed mechanism of plant physiologies regulated by endogenously produced ROS has been reported. ROS were shown to regulate seed germination^60^. Meanwhile, plant hormones such as GAs are sensitively regulated by ROS^61^. ROS also regulate GA levels^53^. GAs are key regulators of plant growth and development in both normal and stressed conditions^61–63^. Endogenous ROS accumulation is important in breaking seed dormancy, and stimulating seed germination^53^, a process that involves GA signaling^61^. ROS produced by nicotinamide adenine dinucleotide phosphate hydrogen (NADPH) oxidases promote GA biosynthesis in embryos, in which GA enhances NADPH oxidases and ROS levels in aleurone cells to induce α-amylase^64^. Therefore, as the reciprocal regulations of GA and ROS involve positive regulation in seed germination^53,61,64^, and increased GA levels can overcome photocatalytic NP-derived oxidative stress^65^, reasonably observed the TiO_2_ photocatalysis-mediated ROS-dependent enhancement of seed germination (Figs. 6, 7). Despite this observation, exogenous ROS produced by photocatalytic NPs are generally associated with an impression of biocides, with negative impacts on lifeforms^8,12,66^. The finding that environmental ROS can actively participate in the delicate regulation of the plant physiologies surprised us and may imply ancient environment–plant interplay, particularly because these photocatalytic NPs have existed on Earth as natural NPs for hundreds of millions of years.

TiO_2_ NPs are not only present in forms of ENPs, but also naturally; for example, naturally formed soil and rock on the earth contain various levels of TiO_2_ with a range of 0.1 to − 1.5% of total weight^28–31^. Some volcanic ash, rock (basalt) and andisol samples were shown to contain high TiO_2_ levels with over 2% of total weight^67,68^. Volcanic ash exerts good fertilization property to enhance plant growth ^69^; besides the ability to function as a supplement of essential and rare elements, our observation suggested that the TiO_2_ content may partly contribute to the enhancement of plant growth. This hypothesis and relevant mechanism are worthy of further investigation.

Our data revealed that the supplements of photocatalytic TiO_2_ and ZnO NPs in the soil induced growth of both shoot and root parts of the seedlings, in which the shoot growth was more pronounced than root growth (Figs. 2, 4). GAs are known to enhance shoot growth^63^, so supplements of photocatalytic TiO2 NPs somehow enhanced the GA levels (Fig. 7C). The shoots will grow into leaves, and which are the major parts used by consumers of these leafy vegetables. The supplementation of photocatalytic NPs could be a novel approach to enhance the growth of agricultural plants with economic value. In addition, new technological advancements such as green synthesis have shown great potentials of ENPs in sustainable agriculture^7,10,11,70^. However, the use of these photocatalytic NPs must be controlled in a proper level. As the data revealed in this study (Figs. 2, 4), the photocatalytic NPs played a dual role in seed growth. Given that excessive ROS can be detrimental, once the level of NPs is higher than an acceptable range, the plant-growth enhancing effect will turn into a toxic effect (Figs. 2, 4). Accordingly, overuse of the photocatalytic NPs on plant fertilization should be avoided to prevent plant toxicity and increased the environmental burden. As ENPs are produced with increasing amount worldwide, the environmental photocatalytic NP levels have become a critical factor for the survival of natural and agricultural plants. For sustainable development and environmental health, monitoring of the accumulation and turnover rates of photocatalytic NPs in the soil is critical.

## Conclusion

In summary, we found that the photocatalytic activity of TiO_2_ NPs positively affected seed germination and growth through gibberellins in a plant-tolerable range (0.1 and 0.2 mg/cm^2^), whereas overdosing (1 mg/cm^2^) induced tissue damage. This positive effect of TiO_2_ NPs involved photocatalysis-elicited ROS and GA regulation; as treatments of antioxidant NAC can suppress both TiO_2_-mediated enhancements on enhance the seed germination and seedling growth and TiO_2_-mediated up-regulation of GA levels. Accordingly, supplementation of photocatalytic NPs could be a theoretically feasible approach in the development of sustainable plant-growth enhancing agents. On the other hand, as treatments of high doses of photocatalytic NPs can strongly suppress the seedling growth, photocatalytic NPs can also be used as herbicides. Because these photocatalytic NPs have dual (low dose: enhancing; high dose: suppressing) roles, future studies focused on the maintenance of these NPs in a desired level in the soil could be important for sustainable development and environmental health.

## Supplementary Information

Supplementary Table S1.

## Data Availability

The datasets generated and analyzed during the current study are not publicly available due to potential patent filing after the report, but are available from the corresponding author on reasonable request.
