# Supplementary material for: Exposure to low levels of photocatalytic TiO2 nanoparticles enhances seed germination and seedling growth of amaranth and cruciferous vegetables
Source: Sci Rep. 2022 Oct 29;12:18228. doi: 10.1038/s41598-022-23179-9 (PMC9617883; doi:10.1038/s41598-022-23179-9)
Supplement: Supplementary file 1 — Supplementary Table S1. [file 41598_2022_23179_MOESM1_ESM.docx]

**Supplemental information for:**

**Exposure to low levels of photocatalytic TiO_2_ nanoparticles enhances seed germination and seedling growth of amaranth and cruciferous vegetables**

Chi-Cheng Li ^1, 2^ Sian-Ming Jhou ^3, 4†^, Yi-Chen Li ^3, 4†^, Jhih-Wei Ciou ^3, 4†^, You-Yen Lin ^3^, Shih-Che Hung ^3,5^, Jen-Hsiang Chang ^6^, Jen-Che Chang ^7^, Der-Shan Sun ^3, 5^, Ming-Lun Chou ^8^, and Hsin-Hou Chang ^3, 5^*

^1^ Department of Hematology and Oncology, Buddhist Tzu Chi General Hospital, Hualien, Taiwan.

^2^ Center of Stem Cell & Precision Medicine, Hualien Tzu Chi Hospital, Hualien, Taiwan.

^3^ Department of Molecular Biology and Human Genetics, Tzu-Chi University, Hualien, Taiwan.

^4^ Tzu-Chi Senior High School Affiliated with Tzu-Chi University, Tzu-Chi University, Hualien, Taiwan.

^5^ Institute of Medical Sciences, Tzu-Chi University, Hualien, Taiwan.

^6^ Department and Graduate School of Computer Science, National Pingtung University, Pingtung, Taiwan.

^7^ Stella Maris High School, Hualien, Taiwan.

^8^ Department of Life Sciences, Tzu-Chi University, Hualien, Taiwan.

Running title: Photocatalytic-NPs enhanced seed germination and growth

Key words: seed germination, TiO_2_, ZnO, nanoparticle, reactive oxygen species, gibberellins

† These authors contributed equally to this work.

* To whom correspondence and reprint requests should be addressed.

Hsin-Hou Chang Ph.D.

Room D407, Tzu-Chi University, No. 701, Section 3, Chung-Yang Road, Hualien 97004, Taiwan.

Tel: 886-3-8565301 ext 2667. Fax: 886-3-8578386.

E-mail: [hhchang@mail.tcu.edu.tw](mailto:hhchang@mail.tcu.edu.tw)

**Table S1**

**List of abbreviations (in order of appearance)**

Titanium dioxide (TiO_2_)

Nanoparticles (NPs)

Engineered NPs (ENPs)

Ultraviolet (UV)

N-acetylcysteine (NAC)

Zinc oxide (ZnO)

Single-walled carbon nanotubes (CNTs)

Nanoparticle of single-walled carbon nanotube (CNT-NP)

Nanodiamond (ND)

Nanoparticle of nanodiamond (ND-NP)

Silicon dioxide (SiO_2_)

Silicon dioxide nanoparticle (SiO_2_-NP)

Light-emitting diode (LED)

Gibberellic acids (GAs)

Enzyme-linked immunosorbent assay (ELISA)

Phosphate-buffered saline (PBS)

Transmission electron microscopy (TEM)

Scanning electron microscopy (SEM)

Analysis of variance (ANOVA)

Reactive oxygen species (ROS)

2-dimensional (2D)

Carbon-containing TiO_2_ [TiO_2_(C)]

Platinum-containing TiO_2_ [TiO_2_(Pt)]

Nicotinamide adenine dinucleotide phosphate hydrogen (NADPH)
